# High Glucose Induces CCL20 in Proximal Tubular Cells via Activation of the KCa3.1 Channel

**DOI:** 10.1371/journal.pone.0095173

**Published:** 2014-04-14

**Authors:** Chunling Huang, Carol A. Pollock, Xin-Ming Chen

**Affiliations:** 1 Kolling Institute of Medical Research, Royal North Shore Hospital, Sydney Medical School, University of Sydney, Sydney, NSW, Australia; 2 Xiamen Center of Clinical Laboratory, Xiamen Zhongshan Hospital, Medical College of Xiamen University, Xiamen, China; National Center for Scientific Research Demokritos, Greece

## Abstract

**Background:**

Inflammation plays a key role in the development and progression of diabetic nephropathy (DN). KCa3.1, a calcium activated potassium channel protein, is associated with vascular inflammation, atherogenesis, and proliferation of endothelial cells, macrophages, and fibroblasts. We have previously demonstrated that the KCa3.1 channel is activated by TGF-β1 and blockade of KCa3.1 ameliorates renal fibrotic responses in DN through inhibition of the TGF-β1 pathway. The present study aimed to identify the role of KCa3.1 in the inflammatory responses inherent in DN.

**Methods:**

Human proximal tubular cells (HK2 cells) were exposed to high glucose (HG) in the presence or absence of the KCa3.1 inhibitor TRAM34 for 6 days. The proinflammatory cytokine chemokine (C-C motif) ligand 20 (CCL20) expression was examined by real-time PCR and enzyme-linked immunosorbent assay (ELISA). The activity of nuclear factor-κB (NF-κB) was measured by nuclear extraction and electrophoretic mobility shift assay (EMSA). *In vivo,* the expression of CCL20, the activity of NF-κB and macrophage infiltration (CD68 positive cells) were examined by real-time PCR and/or immunohistochemistry staining in kidneys from diabetic or KCa3.1-/- mice, and in eNOS-/- diabetic mice treated with the KCa3.1 channel inhibitor TRAM34.

**Results:**

*In vitro* data showed that TRAM34 inhibited CCL20 expression and NF-κB activation induced by HG in HK2 cells. Both mRNA and protein levels of CCL20 significantly decreased in kidneys of diabetic KCa3.1-/- mice compared to diabetic wild type mice. Similarly, TRAM34 reduced CCL20 expression and NF-κB activation in diabetic eNOS-/- mice compared to diabetic controls. Blocking the KCa3.1 channel in both animal models led to a reduction in phosphorylated NF-κB.

**Conclusions:**

Overexpression of CCL20 in human proximal tubular cells is inhibited by blockade of KCa3.1 under diabetic conditions through inhibition of the NF-κB pathway.

## Introduction

The intermediate-conductance calcium-activated potassium channel KCa3.1 (also known as IK1, SK4 or KCNN4) is a member of the calcium-activated potassium channel (KCa) family. KCa3.1 regulates K^+^ efflux, increasing the driving force for Ca^2+^ entry through hyperpolarization of the plasma membrane [1]. It has been shown that KCa3.1-mediated Ca^2+^ influx is associated with inflammation, atherogenesis and proliferation of endothelial cells, T lymphocytes, macrophages and fibroblasts [2]–[6]. Therefore, KCa3.1 is a potential molecular target for pharmacological intervention in vascular restenosis, urinary incontinence, prostate cancer, and autoimmune disease [7]–[9].

DN is increasingly considered as an inflammatory disease characterized by macrophage infiltration [10]. Inflammatory chemokines have been shown to play a key role in the development of DN. Various factors of diabetic milieu can induce renal expression of chemokines and thereby mediate the macrophage responses that ultimately cause renal injury. Evidence from renal biopsies and STZ-induced diabetic animal models have shown that macrophage accumulation in diabetic kidneys is associated with declining renal function [11]. Chemokine (C-C motif) ligand 20 (CCL20) also known as macrophage inflammatory protein-3α, has been reported to be expressed in epithelial cells, endothelial cells and fibroblasts in many organs [12], [13]. The human CCL20 gene was mapped to chromosome 2q33–37 and its promoter region contains possible binding sites for NF-κB which are known to be involved in the transcriptional regulation of various inflammatory cytokines and chemokines [14]. Our group has previously identified a significantly increased level of CCL20 in the HG-induced renal proximal tubule cells and in the kidney of diabetic rats, indicating that CCL20 is involved in the pathogenesis of DN [15]. Thus, any agent(s) with anti-inflammatory activities to lower inflammatory cytokines like CCL20 may potentially prevent or delay the development of diabetic renal injury. Recently, we have demonstrated that blockade of KCa3.1 ameliorates renal fibrosis in diabetic mice through inhibition of the TGF-β1 signaling pathway [16]. However, the centrality of KCa3.1 activation to HG induced inflammation remains unknown.

In this study we investigated CCL20 in proximal tubular cells exposed to HG with or without TRAM34 in vitro and the role of KCa3.1 in the inflammatory responses in DN using two STZ-induced diabetic mice models. Our results demonstrate that blockade of KCa3.1 was able to attenuate the upregulation of CCL20 expression and macrophage infiltration induced by diabetes, which is mediated through inhibition of NF-κB activation.

## Material and Methods

### Cell culture

HK2 cells were grown in keratinocyte serum-free medium (Invitrogen, CA). The cells were exposed to HG (25 mM) in the presence or absence of TRAM34 (4 uM) [17] for 6 days. In all experiments, cells were serum starved overnight before adding HG and TRAM34. To evaluate the effect of NF-κB inhibitor on CCL20 expression, HK2 cells were exposed to the NF-κB inhibitor pyrrolidine dithiocarbamate (PDTC) (25 µM, sigma) [18] during incubation with HG (25 mM) for 6 days.

### Ethics Statement

Experimental procedures adhered to the guidelines of the National Health and Medical Research Council of Australia's Code for the Care and Use of Animals for Scientific Purposes and were approved by the Animal Research Ethics Committee of Royal North Shore Hospital.

### Animal studies

KCa3.1-/- mice were kindly provided by Dr. James Melvin (National Institute of Dental and Craniofacial Research, Bethesda, MD, USA). As described before [16], [19], A group of eight-week-old male KCa3.1+/+ (C57B/6) mice (n = 8), KCa3.1-/- mice (n = 8) and eNOS-/-mice (n = 6) (Jackson laboratory, ME) weighing approximately 20 to 25 g were assigned to receive either 55 mg/kg of streptozotocin (STZ) (Sigma, MO) diluted in 0.1 M citrate buffer, pH 4.5, or citrate buffer alone by intraperitoneal injection for five consecutive days as described previously [20]. STZ-treated animals with blood glucose >16 mmol/l were considered diabetic. Those KCa3.1+/+ and eNOS-/-mice received citrate buffer alone served as non-diabetic controls. eNOS-/- diabetic mice were then randomized into two groups, receiving treatment with TRAM34, 120 mg/kg/day intraperitoneally or vehicle (DMSO) alone for 24 weeks. Treatment commenced within 24 h of the last STZ injection. All animals were housed in the Kearns Animal Facility of Kolling Institute of Medical Research with a stable environment maintained at 22±1°C with a 12/12-h light-dark cycle. After animals were culled, left kidneys were removed and snap frozen for the isolation of RNA or protein, and right kidneys were perfused with PBS and fixed in 10% buffered formalin for histological examination.

### EMSA for NF-kB activity

Nuclear extracts were prepared using a NucBuster Protein Extraction Kit (Novagen, Germany) in accordance with the manufacturer's instruction. Nuclear proteins were quantitated by BCA assay (Pierce, Waltham). EMSA were performed using the DIG Gel Shift Kit (Roche Applied Science, Indianapolis) with 20 µg of nuclear extract from each treatment condition. Nuclear extract were incubated with 1 µg poly [d (I-C)] as the nonspecific competitor, 1 µg poly L-lysine in a binding buffer [400 mM KCl, 80 mM HEPES, 2 mM DTT, 0.8 mM EDTA, 80% glycerol, pH 8)] and digoxygenin (dig)-labeled NF-κB (5′-AGTTGAGGGGACTTTCCCAGGC-3′) consensus oligonucleotide (Promega, Wisconsin) for 30 min at room temperature. The reaction mixture was electrophoresed through 6% polyacrylamide gels, transferred onto nylon positively charged membrane (Roche Applied Science, Indianapolis), and then cross-linked using a UV-transilluminator for 3 min. The membrane was subjected to immunological detection using an anti-dig-AP conjugate and chemiluminescence. Results were analyzed using Image J software.

### RNA isolation and RT-PCR analysis

Total RNA was extracted using GenElute Mammalian Total RNA Miniprep Kit (Sigma) for cell samples or Trizol (Invitrogen) for tissue samples. The cDNA was synthesized using SuperScript VILO cDNA Synthesis Kit (Invitrogen, CA). Quantitative real-time PCR was performed using the SYBR Green PCR master mix kit (Invitrogen, CA) with the intron-spanning primers on ABI-Prism-7900 Sequence Detection System (Applied Biosystems). The PCR primer sets for human Glyceraldehyde 3-phosphate dehydrogenase (GAPDH): (forward) 5′-AGCCACATCGCTCAGACAC-3′; (reverse) 5′-GCCCAATACGACCAAATCC-3′, human CCL20: (forward) 5′-AGAGT TTGCTCCTGGCTG-3′; (reverse) 5′-GGATGAAGAATACGGTCTGTG-3′, mice CCL20: (forward) 5′-CTTGCTTTGGCATGGGTACT-3′; (reverse) 5′-GAGGCAACA GTCGTAGTTGCT-3′, mice CD68: (forward) 5′- GACCTACATCAGAGCCCGAGT-3′, (reverse) 5′-CGCCATGAATGTCCACTG-3′ and mice β-actin: (forward) 5′-TGACAGGATGCAGAAGGAGA-3′, (reverse) 5′-CGCTCAGGAGGAGCAATG-3′. The reaction conditions consisted of 1 cycle of initial denaturation at 95°C for 5 min, followed by 45 cycles of 95°C for 15 seconds and 60°C for 1 min. Reaction specificity was confirmed by melting curve analysis. For quantitative analysis, relative mRNA levels were calculated according to the 2^−ΔΔCt^ method.

### ELISA for CCL20 protein expression

The levels of secreted CCL20 under different experimental conditions were determined in conditioned culture media using commercial ELISA kits (R&D Systems, Minneapolis) according to the manufacturer's protocol. All procedures were performed at room temperature, and the mean absorbance of the standards and samples were detected in duplicate. Briefly, culture medium was added to a 96-well microplate coated with monoclonal antibody specific for human CCL20 for 2 h. After careful wash, CCL20 conjugate was added and incubated for another 2 h followed by streptavidin-Peroxidase enzyme. After a further incubation for 30 minutes at room temperature and repeated washes, color reagents were added. The intensity of the color was measured by microplate reader at 450 nm. Each protein level was normalized with cell lysis.

### Histology and immunohistochemistry

Paraffin-embedded or frozen kidney sections were used for immunohistochemical staining [21]. Briefly, endogenous peroxidase activity was blocked by incubation in 0.3% hydrogen peroxide. After pre-incubation with 10% protein block (Dako, CA) for 10 minutes at room temperature to block nonspecific binding of antibodies, the tissues were incubated overnight at 4°C with primary antibodies against CCL20 (Lifespan Bioscience, WA, paraffin tissues), CD68 (AbD Serotec Oxford, UK, frozen tissues) and phosphorylated NF-κB-P65 (Santa Cruz Biotechnology, CA, paraffin tissues). After incubation with appropriate secondary antibodies, sections were developed with 3, 3-diaminobenzidine (Dako, CA) to produce a brown colour and counterstained with haematoxylin. Positive signals in the renal cortex regions were quantified using Image J software as previously described [22]. The number of cells positive for CD68+ or phosphorylated NF-κB-P65+ was counted in 10 high power fields (×400) of the tubulointerstitium.

### Statistical analysis

Results from at least three independent experiments were expressed as mean ± SEM. Statistical analysis of data from two groups was compared by student two-tailed t-test. Data from multiple groups was performed by one-way ANOVA, followed by Tukey post test. Statistical significance was determined as *P*<0.05.

## Results

### KCa3.1 blocker TRAM34 inhibited CCL20 expression and NF-κB activation induced by HG in human renal proximal tubular cells

**Figure 1:**
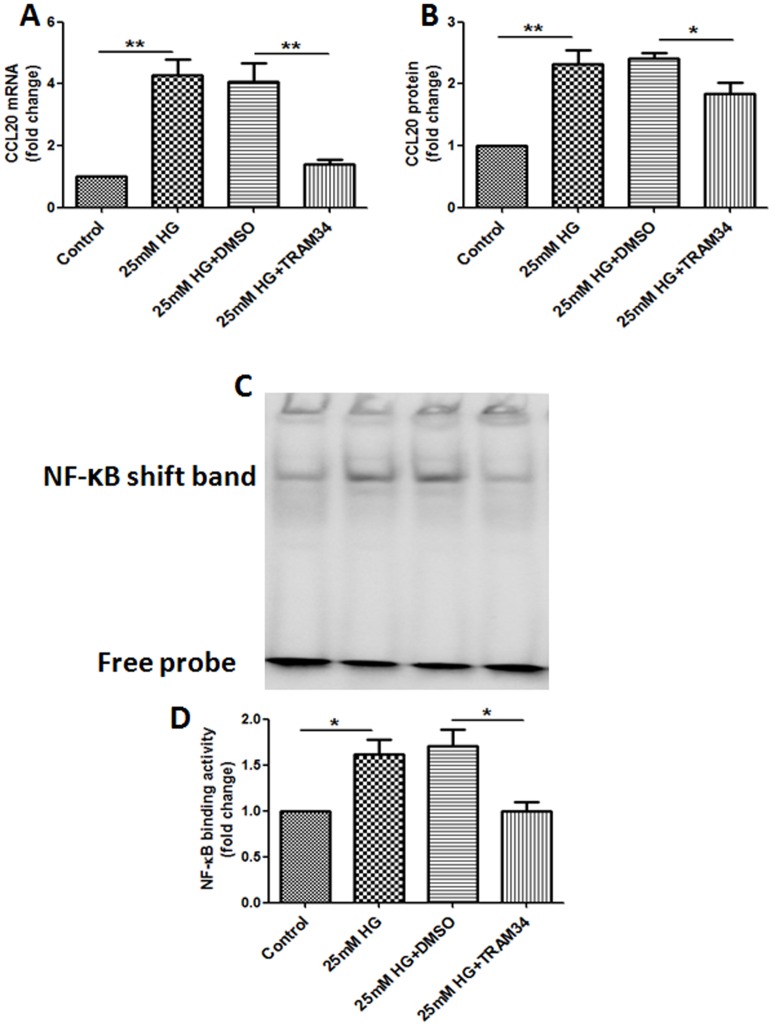
KCa3.1 blocker TRAM34 inhibited CCL20 synthesis and NF-κB activation induced by HG in human renal proximal tubular cells. HK2 cells were treated with either control, HG (25 mM) or high glucose (25 mM) combined with DMSO (vehicle control) or TRAM34 (4 µM) for 6 days. (A) RT-PCR results showed that TRAM34 inhibited HG-induced CCL20 mRNA expression. (B) ELISA result showed that TRAM34 inhibited the induction of CCL20 in HG treated HK2 cells. Nuclear extracts of those cells were used to test NF-κB binding activity by EMSA. (C) EMSA results showed that TRAM34 inhibited HG-induced NF-κB activation in HK2 cells. (D) The quantitive result for NF-κB binding acivity. Results are presented as means ± SEM. *P<0.05 and **P<0.01, n = 3.

To determine the effects of KCa3.1 on the synthesis of CCL20 in HG-stimulated HK2 cells, HK2 cells exposed to HG were concurrently incubated with TRAM34. As expected, exposure of HK2 cells to HG resulted in significantly increased expression of CCL20 compared with control, while concurrent exposure to TRAM34 significantly inhibited HG-induced mRNA and protein expression of CCL20 (*P*<0.05, Fig.1A and B) in HK2 cells. NF-κB signaling pathway is activated in the diabetic kidney and has been shown to be an important transcription factor involved in the pathophysiology of DN [23]. We therefore investigated whether NF-κB is involved in KCa3.1 mediated CCL20 expression in HG-stimulated HK2 cells. NF-κB-DNA binding affinity was examined by EMSA. As shown in Fig.1C and D, HG significantly increased NF-κB-DNA binding activity compared with control which was reversed by treatment with TRAM34 in HK2 cell (*P*<0.05).

### Upregulation of CCL20 expression in HG-stimulated HK2 cells was inhibited by NF-κB inhibition

**Figure 2:**
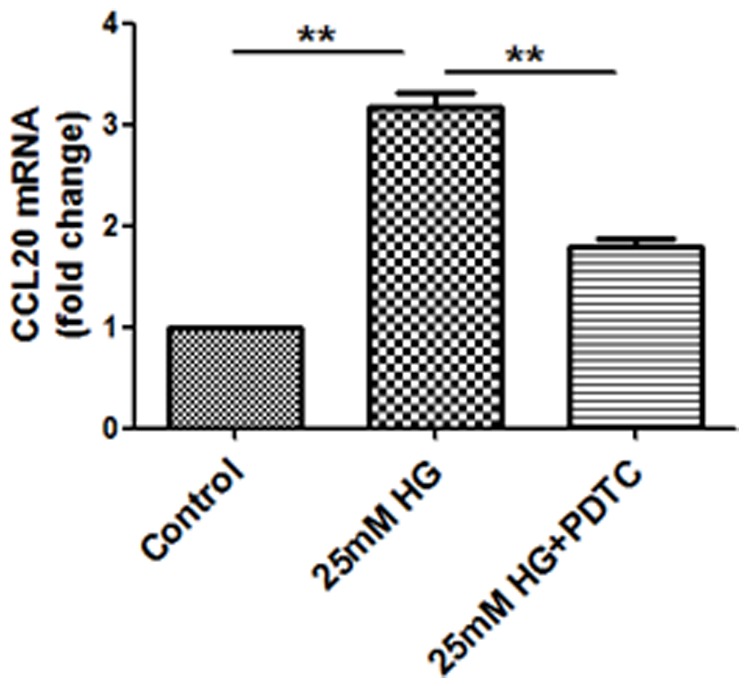
Upregulation of CCL20 expression in HG-stimulated HK2 cells was inhibited by NF-κB inhibition. HK2 cells were treated with either control, HG (25 mM) or HG (25 mM) combined with NF-κB inhibitor PDTC (25 µM) for 6 days. RT-PCR results showed that PDTC inhibited HG-induced CCL20 mRNA expression in HK2 cells. Results are presented as means ± SEM. ** *P*<0.01, n = 3.

To further confirm whether upregulation of CCL20 expression in response to HG was dependent on NF-κB activation, we studied the expression of the chemokine in proximal tubular cells exposed to HG in the presence of the NF-κB inhibitor PDTC (25 µM). As shown in Fig.2, PDTC reduced by more than 40% the increase in CCL20 transcript levels in response to HG stimulation (*P*<0.05). These results confirm the involvement of the NF-κB pathway in HG-induced CCL20 expression.

### Blockade of KCa3.1 suppressed CCL20 expression in diabetic mice

**Figure 3:**
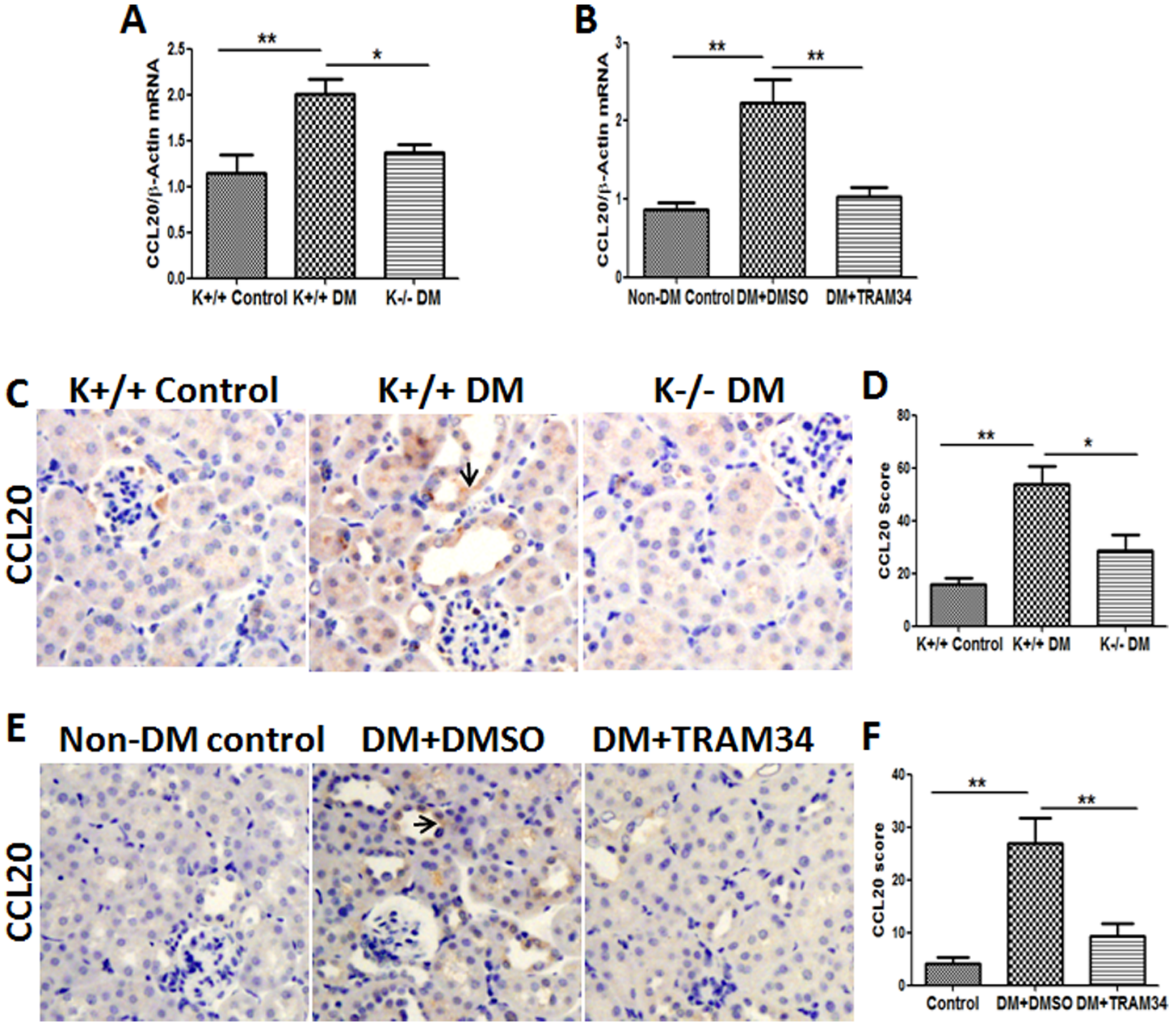
Blockade of KCa3.1 suppressed CCL20 expression in diabetic mice. Quantitative RT-PCR showed increased mRNA levels of CCL20 (A), in the kidneys of diabetic KCa3.1+/+ mice but reversed in diabetic KCa3.1-/- mice (n = 8). Quantitative RT-PCR showed increased mRNA expression of CCL20 (B) in the kidneys of diabetic eNOS-/- mice compared to control mice but reduced with TRAM34 treatment (DM+TRAM34) (n = 6). Representative images (C) show immunohistochemical staining of CCL20 in the renal cortex from control KCa3.1+/+ mice, diabetic KCa3.1+/+ mice and diabetic Kca3.1-/- mice (n = 8). Representative images (E) show immunohistochemical staining of CCL20 in the renal cortex from control mice, diabetic eNOS-/- mice and diabetic eNOS-/- mice treated with TRAM34. (D, F) The quantitation of CCL20 expression in mice kidneys. Results are presented as mean ± SEM. *P<0.05 and **P<0.01. Original magnification: ×200.

To support the in vitro finding, we next sought to determine the role of KCa3.1 in CCL20 expression using two *in vivo* models of DN as described above. Real-time PCR analyses of kidney tissues revealed that the expression of CCL20 was increased by 1.8-fold in diabetic KCa3.1+/+ group, and was reduced in the diabetic KCa3.1-/- group (*P*<0.05, Fig.3A). Immnunohistochemistry analysis demonstrated a significant reduction of CCL20 in diabetic kidneys of KCa3.1-/- mice as compared with diabetic KCa3.1+/+ controls (*P*<0.01, Fig. 3C and D). Consistent with this finding, we also observed a significant decrease in CCL20 expression in the kidneys of diabetic eNOS-/- mice treated with the KCa3.1 blocker TRAM34 compared to the diabetic control group (*P*<0.05, Fig. 3B, E and F).These data suggest that KCa3.1 contributes to the production of CCL20 in DN.

### Blockade of KCa3.1 prevented diabetes-induced macrophage infiltration into kidney

**Figure 4:**
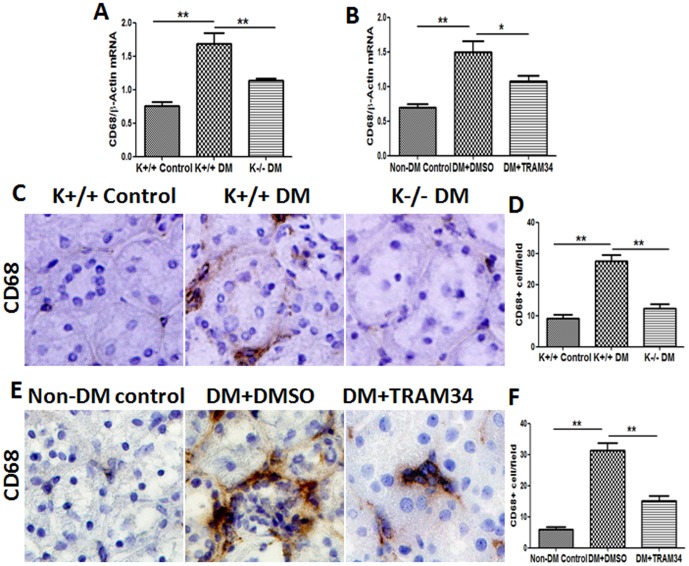
Blockade of KCa3.1 prevented diabetes-induced macrophage infiltration into Kidney. (A) Quantitative RT-PCR showed increased mRNA levels of CD68 in the kidneys of diabetic KCa3.1+/+ mice but reversed in diabetic KCa3.1-/- mice (n = 8). (B) Quantitative RT-PCR showed increased mRNA levels of CD68 in the kidneys of diabetic eNOS-/- mice but reversed in diabetic eNOS-/- mice treated with TRAM34 (n = 6). (C) Immunohistochemical analysis showed increased CD68 in diabetic KCa3.1+/+ kidneys compared to control mice and reversed expression of CD68 in diabetic KCa3.1-/- kidneys (n = 8). (E) Immunohistochemical analysis showed increased CD68 in diabetic eNOS-/- kidneys compared to control mice and reversed expression of CD68 in diabetic eNOS-/- kidneys treated with TRAM34 (n = 6). (D, F) The quantitation of CD68 expression in mice kidneys. Results are presented as mean ± SEM. *P<0.05 and **P<0.01. Original magnification: ×400.

To characterize the role of KCa3.1 in the regulation of macrophage infiltration, we examined one macrophage marker CD68 in kidney tissues. As indicated in Fig. 4A, a marked induction of CD68 (2.2-fold, *P*<0.01) mRNA was observed in the kidneys of diabetic KCa3.1+/+ mice when compared with non-diabetic controls. KCa3.1 deficiency significantly inhibited the expression of CD68 in diabetic kidneys. In addition, histopathological analysis of renal cross-sections demonstrated a 65% reduction of CD68 expression in diabetic kidneys of KCa3.1-/- mice as compared with diabetic KCa3.1+/+ controls (*P*<0.01, Fig. 4C and D). Consistently, we also observed a significant decrease in CD68 expression in the kidneys of diabetic eNOS-/- mice treated with the KCa3.1 blocker TRAM34 compared to the diabetic control group (*P*<0.05, Fig. 4B, E and F).

### Blockade of KCa3.1 reversed diabetes-induced NF-κB activation in diabetic kidneys

**Figure 5:**
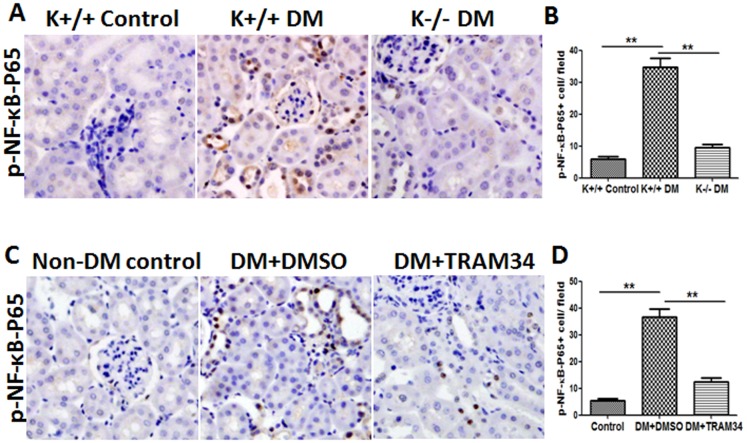
Blockade of KCa3.1 reversed diabetes-induced NF-κB activation in diabetic kidneys. (A) Immunohistochemical analysis showed increased phosphorylated NF-κB-P65 in diabetic KCa3.1+/+ kidneys compared to control mice and reversed activation of NF-κB in diabetic KCa3.1-/- kidneys (n = 8). (C) Immunohistochemical analysis showed increased NF-κB activation in diabetic eNOS-/- kidneys compared to control mice and reversed activation of NF-κB in diabetic eNOS-/- kidneys treated with TRAM34 (n = 6). (B, D) The quantitation of phosphorylated NF-κB expression in mice kidneys. Results are presented as mean ± SEM. *P<0.05 and **P<0.01. Original magnification: ×200.

To further determine whether diabetes-induced activation of NF-κB is mediated by KCa3.1, NF-κB activation was examined by immunohistochemical staining of phosphorylated NF-κB-P65 in experimental mice kidney tissues. As shown in Fig. 5A and B, NF-κB signaling was strongly activated in diabetic KCa3.1+/+ mice compared with control mice. However, the activation was inhibited in KCa3.1-/- diabetic mice (*P*<0.01). Similarly, enhanced activation of NF-κB was suppressed in diabetic kidneys from TRAM34-treated mice compared with vehicle-treated mice (*P*<0.01, Fig.5C and D). Taken together, these data indicate that KCa3.1 mediated expression of CCL20 may occur through NF-κB signaling pathway in the diabetic kidneys.

## Discussion

In the current study, we demonstrate that blockade of KCa3.1 inhibited hyperglycemia induced up-regulation of CCL20 in both in vitro and in vivo models. In addition, our results showed that increased CCL20 expression was accompanied by increasing macrophage infiltration into the tubulointerstitium, indicating that CCL20 is associated with renal macrophage infiltration in DN. Moreover, the findings suggest that the anti-inflammatory effects of KCa3.1 inhibition are mediated by a reduction in CCL20 and NF-κB activity. Taken together, these findings suggest that KCa3.1 plays a role in inflammation and that the KCa3.1 inhibitor TRAM34 exerts anti-inflammatory effects via inhibition of the NF-κB signaling pathway.

Accumulating data from clinical and animal studies have indicated that inflammatory cytokines play a critical role in the development and progression of DN [24], [25]. CCL20 has been identified as having a key role in the development of DN. CCL20 is produced by activated cells in inflamed tissues [26], and its expression can be induced by cytokines such as TGF-β1 thus contributing to various inflammatory disease states [27]. Woltman et al have demonstrated that CCL20 accumulates in the kidney tubules and urine of patients undergoing renal transplant rejection, suggesting CCL20 plays a critical role in kidney inflammation [28]. Studies have also reported that CCL20 is increased in pancreatic beta-cells undergoing inflammatory insults in developing diabetes [29]. Recent studies by our group further confirm that CCL20 is a significant pathogenic mediator in DN [30]. Targeting pro-inflammatory chemokine CCL20 will provide novel strategy to treat diabetic kidney disease. Ca2+-activated K+ channels can communicate directly from Ca2+ signal pathways to changes in membrane potential that are critically required for various cellular processes. It has been shown that KCa3.1-mediated elevation of intracellular calcium is necessary for the production of inflammatory chemokines and cytokines by T cells, macrophages and mast cells [31], [32]. The contribution of KCa3.1 to pathophysiological inflammatory can be further highlighted by the role of the channel in vascular diseases, particularly in restenosis and atherosclerosis [33], [34]. Indeed we have previously demonstrated that the KCa3.1 channel activity is upregulated by TGF-β1 in kidney tubular cells [35]. In this study, we observed that blockade of KCa3.1 inhibited HG induced CCL20 mRNA and protein expression, which correlates with the downregulation of macrophage number indicated by CD68. These results suggest that inhibition of KCa3.1 may play a protective role against DN by suppressing CCL20 expression and macrophage infiltration.

NF-κB is a family of pleiotropic transcription factors which induces or represses genes by binding to discrete DNA sequences known as κB elements in promoter and enhancer elements of target genes [36]. Diabetic pathological stress, like hyperglycemia, can stimulate the activation of NF-κB pathways, which evoke the excessive production of inflammatory, oxidative, and even fibrotic molecules and promote the pathogenesis of DN. NF-κB activation has been demonstrated in renal biopsy from patient and renal cortical tissue of animals with DN, indicating that NF-κB is an important transcription factor involved in the pathophysiology of DN [37], [38]. Therefore, several treatments in current use are associated with actions on NF-κB pathway. For instance, studies have shown that the renoprotective effects of thiazolidinediones are postulated to be at least partly related to inhibition of NF-κB activation [39]. In this study, we observed that the activation of NF-κB in renal tissue was significantly increased in untreated diabetic mice, which was markedly reduced by the inhibition of KCa3.1. This finding is supported by our in vitro results, demonstrating that blockade of KCa3.1 suppressed HG-induced increased NF-κB binding activity.

In summary, the present study demonstrated that blockade of KCa3.1 significantly suppressed CCL20 expression as well as inhibited macrophage accumulation in HG-induced proximal tubular cells and STZ-induced diabetic mice. The anti-inflammatory effect of KCa3.1 inhibitor on DN was closely associated with suppressing the activation of the NF-κB-mediated inflammatory pathway. These in vitro and in vivo studies provide a basis for further exploring the therapeutic potentials of KCa3.1 in the intervention and prevention of DN.
